# Vitamin D Deficiency Promotes Liver Tumor Growth in Transforming Growth Factor-β/Smad3-Deficient Mice Through Wnt and Toll-like Receptor 7 Pathway Modulation

**DOI:** 10.1038/srep30217

**Published:** 2016-07-26

**Authors:** Jian Chen, Lior H. Katz, Nina M. Muñoz, Shoujun Gu, Ji-hyun Shin, Wilma S. Jogunoori, Mi-Hye Lee, Mitchell D. Belkin, Sang-Bae Kim, Jon C. White, Jaclyn Andricovich, Alexandros Tzatsos, Shulin Li, Sang Soo Kim, Kirti Shetty, Bibhuti Mishra, Asif Rashid, Ju-Seog Lee, Lopa Mishra

**Affiliations:** 1 The University of Texas MD Anderson Cancer Center, Department of Gastroenterology, Hepatology, and Nutrition, Houston, TX 77030, USA; 2 Sheba Medical Center, Department of Gastroenterology, Tel Hashomer, 52621, Israel; 3 George Washington University, Department of Surgery, Washington, DC, 20052, USA; 4 Institute for Clinical Research, Veterans Affairs Medical Center, Washington, DC, 20422, USA; 5 Georgetown University, Department of Molecular Oncology, Washington, DC, 20057, USA; 6 The University of Texas MD Anderson Cancer Center, Department of Systems Biology, Houston, TX, 77030, USA; 7 George Washington University, Department of Anatomy and Regenerative Biology, Washington, DC, 20052, USA; 8 The University of Texas MD Anderson Cancer Center, Department of Pediatrics, Houston, TX, 77030, USA; 9 National Cancer Center, Radiation Medicine Branch, Goyang, 410-769, Korea; 10 Johns Hopkins University, School of Medicine, Baltimore, MD, 21287, USA; 11 The University of Texas MD Anderson Cancer Center, Department of Pathology, Houston, TX, 77030, USA; 12 Center for Translational Medicine, Department of Surgery and George Washington Cancer Center, George Washington University, Washington, DC, USA

## Abstract

Disruption of the TGF-β pathway is associated with liver fibrosis and suppression of liver tumorigenesis, conditions associated with low Vitamin D (VD) levels. However, potential contributions of VD to liver tumor progression in the context of TGF-β signaling remain unexplored. Our analyses of VD deprivation (VDD) in *in vivo* models of liver tumor formation revealed striking three-fold increases in tumor burden in *Smad3*^+/−^ mice, with a three-fold increase in TLR7 expression compared to controls. ChIP and transcriptional assays confirm Smad3 binding at two *TLR7* promoter SBE sites. Molecular interactions between TGF-β pathway and VDD were validated clinically, where an absence of VD supplementation was associated with low TGF-β pathway member expression levels and β-catenin activation in fibrotic/cirrhotic human liver tissues. Subsequent supplementing VD led to restoration of TGF-β member expression with lower β-catenin levels. Bioinformatics analysis provides positive supportive correlation between somatic mutations for VD-related genes and the TGF-β pathway. We conclude that VDD promotes tumor growth in the context of Smad3 disruption, potentially through regulation of TLR7 expression and β-catenin activation. VD could therefore be a strong candidate for liver cancer prevention in the context of aberrant Smad3 signaling.

Vitamin D (VD) is a major regulator of calcium homeostasis in the body and essential for normal mineralization of bone^1^. The active metabolite of VD, calcitriol (1,25(OH)_2_VD3), accomplishes these by binding to the VD receptor (VDR) and functions via both genomic and non-genomic pathways that include TGF-β-regulated genes such as connective tissue growth factor (*Ctgf*) and collagen type I alpha 2 (*Col1a2*)^2^,^3^. Calcitriol has multiple non-calcemic effects, including anti-proliferative, pro-differentiating, anti-inflammatory, and immunomodulatory activities, supporting a role in cancer prevention and treatment trials^1^,^4^. Epidemiological studies indicate that lower levels of VD may be associated with a higher incidence of death for several cancers including hepatocellular cancer, with an inverse association between cancer risks and circulating levels of VD^5^,^6^. Moreover, 90% of liver cancers in the setting of advanced liver disease are characterized by VD deficiency^7^. Thus, while a strong rationale exists for supplementing patients with advanced liver disease with high doses of VD to prevent liver cancer, specific high risk populations that can be targeted have yet to be identified^8^,^9^.

VDR knockout mice spontaneously develop hepatic fibrosis^10^. In turn, VDR ligands inhibit TGF-β-induced liver fibrosis by binding to co-regulated genes such as *Tgfb1*, *Mmp7* and *Ctgf* and reduce Smad3 occupancy at these sites^10^. VD metabolites suppress TGF-β-mediated fibrosis through modulating multiple pro-fibrotic proteins, for instance, lowering collagen I and III expression and raising expression levels of MMP8, a metalloproteinase that degrades collagens^11^. VD and synthetic analogs are also potent cytostatic or apoptotic agents in hepatic malignant cells that express VDR^4^,^12^. In cell culture experiments, TGF-β has been demonstrated to restore growth-inhibitory effects of VD in a VD-resistant cell line^13^. Yet, while VD and its analogs are considered promising anti-fibrotic agents, signaling pathways that are affected by VD analogs in advanced liver disease and dysplastic hepatic lesions or tumors are not well delineated^5^,^12^.

The TGF-β signaling pathway is aberrant in fibrosis as well as in liver and gastrointestinal cancers, with a complex context-dependent role, promoting epithelial mesenchymal transition (EMT), to suppressing and prompting oncogenesis. It is often considered a driving pathway for these specific tumors. TGF-β mediates its effects through type I and type II serine-threonine receptor kinases. Type II receptor mutant mice are more susceptible to liver tumor formation^14^. The ligand-activated TGF-β receptor complex phosphorylates and activates Smads, specifically the receptor-regulated Smad2 and Smad3, which then form a complex with Smad4 and translocate into the nucleus. Activated Smad complexes additionally recruit transcriptional co-activators and co-repressors that regulate a multitude of target genes, leading to complex outcomes that include connective tissue deposition, cell cycle arrest in G1/S phase, induction of apoptosis, immune suppression, as well as tumorigenesis^15^,^16^,^17^. In view of the roles of both VD and the TGF-β pathway in cirrhosis and liver cancer, we sought to determine whether VD deprivation (VDD) exacerbated liver tumorigenesis in our mouse models. Our results indicate that the markedly high tumor burden in Smad3 mutant mice results from aberrant Toll-like receptor (TLR) expression as well as Wnt signaling, and demonstrate functional regulation of TLR7 through Smad3. Remarkably, VD treatment restores levels of TGF-β pathway members and suppresses β-catenin in patients with cirrhosis or hepatocellular cancer. Lastly, our findings of aberrant expression of the TGF-β members in the context of VD levels, led us to examine whether expression levels of VD-related genes correlated with TGF-β superfamily in human liver cancer. Our studies suggest that in specific patient populations with disruption of TGF-β signaling, low VD markers correlate with activation of the Wnt pathway and a high risk of tumorigenesis.

## Results

### A low VD diet promotes tumor formation in *Smad3* ^+/−^ mice

**Figure 1:**
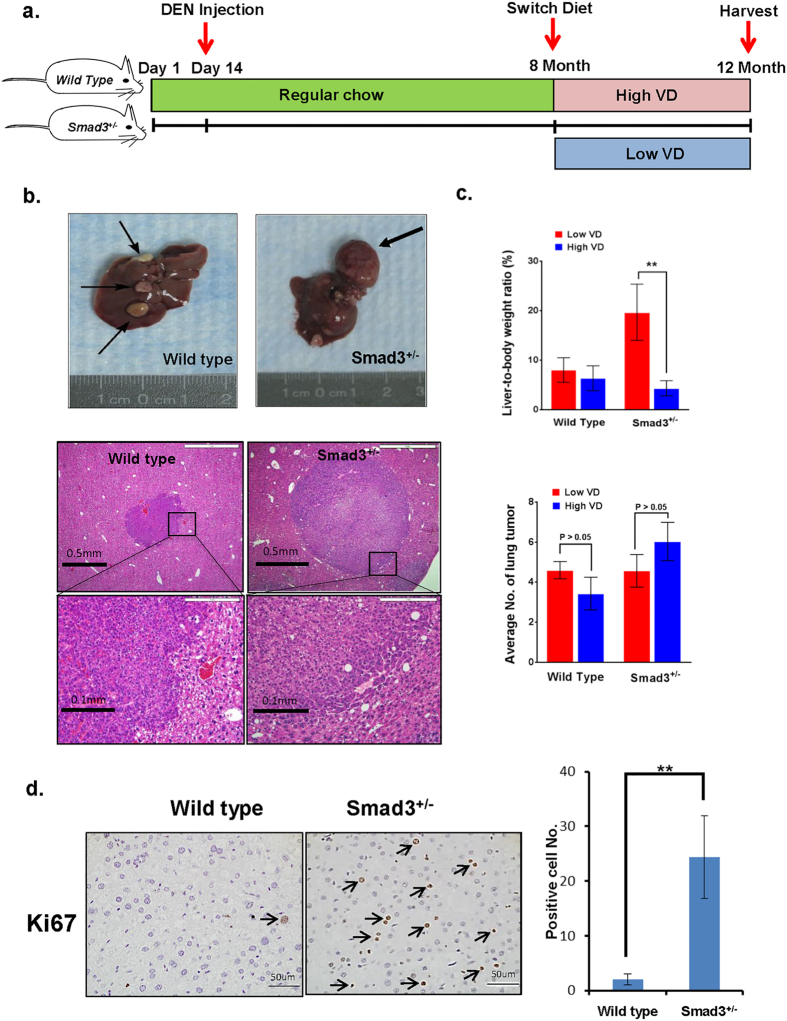
VD deficiency promotes liver tumor growth in the context of Smad3 impairment. (**a**) Diagram of experimental protocol to assess the effects of VD deficiency in *Smad3*^+/−^ mice. Wild type and *Smad3*^+/−^ mice were injected with DEN (50 mg/kg intraperitoneally) at the age of 14 days. All mice were fed a normal VD regimen until 8 months of age and then were switched to either a low VD diet (n = 23) or a high-VD diet (n = 26) for 4 months. (**b**) Upper panels show representative image of liver tumor in wild type and *Smad3*^+/−^ mice with low VD treatment. Compared to wild type control animals, *Smad3*^+/−^ mice fed the VD-deficient diet have larger liver tumors. Lower panels are H&E stains of liver tumor in wild type and *Smad3*^+/−^ mice fed with low VD chow (4×, 20×). (**c**) Liver-to-body weight ratios were measured in both wild type and *Smad3*^+/−^ mice. *Smad3*^+/−^ mice fed the VD-deficient diet had a liver-to-body weight ratio of 18.37%, *Smad3*^+/−^ animals fed the high-VD diet had a ratio of 6.25%. Average number of lung tumors is exhibited as control. High/low VD treatment did not affect the number of lung adenocarcinomas in either *Smad3*^+/−^ or wild type mice. (**d**) Hepatocyte proliferation was analyzed by Ki-67 staining and then quantitatively measured by counting. *Smad3*^+/−^ mice fed the VD-deficient diet had increased cell proliferation compared to wild type control animals. Arrows indicate Ki67 positive nuclei. Results of (**c**,**d**) are presented as mean ± SD. (**p < 0.05, Student’s t-test).

Because a high prevalence of severe VD deficiency has been observed in cirrhosis and liver cancer patients^18^, we examined the role of VD in liver tumor formation in a TGF-β pathway inactivated mouse model. Wild type and *Smad3*^+/−^ mice were injected with the liver carcinogen diethylnitrosamine (DEN) (50 mg/kg) at 14 days of age; then, all mice were fed regular mouse chow. At 8 months, wild type and *Smad3*^+/−^ male mice were fed either a VD-deficient diet (200 IU/kg) (n = 23) or a high-VD diet (10,000 IU/kg) (n = 26) for 4 months. At 1 year, the mice were euthanized, and tumor samples were harvested (Fig. 1a). The representative image shows that *Smad3*^+/−^ mice fed the VD-deficient diet have larger liver tumors compared to wild type control animals (Fig. 1b). Liver tumors were up to 3 times larger in the VD-deprived Smad3 mutants compared to controls. However, average numbers of liver tumors were not altered significantly (Supplementary Table S1). In addition, VD-deprived *Smad3*^+/−^ mice had an average liver-to-body weight ratio of 18.37%, compared to the ratio of 6.25% in *Smad3*^+/−^ animals fed the high-VD diet (Fig. 1c, Supplementary Table S1). We observed increased cell proliferation in VD-deprived *Smad3*^+/−^ mice compared to wild type control animals (Fig. 1d). Because DEN is well known to cause lung cancers in rodents, we therefore sought to analyze the mice for lung cancers as well. Interestingly, high dietary VD/VD-deficient doses did not affect the number of lung adenocarcinomas in either Smad3^+/−^ or wild type mice (Fig. 1c, Supplementary Table S1), and we did not observe a noticeable increase in total tumor burden in the lung at the time the data was collected, suggesting VD levels on tumorigenesis appear to be restricted to the liver.

**Figure 2:**
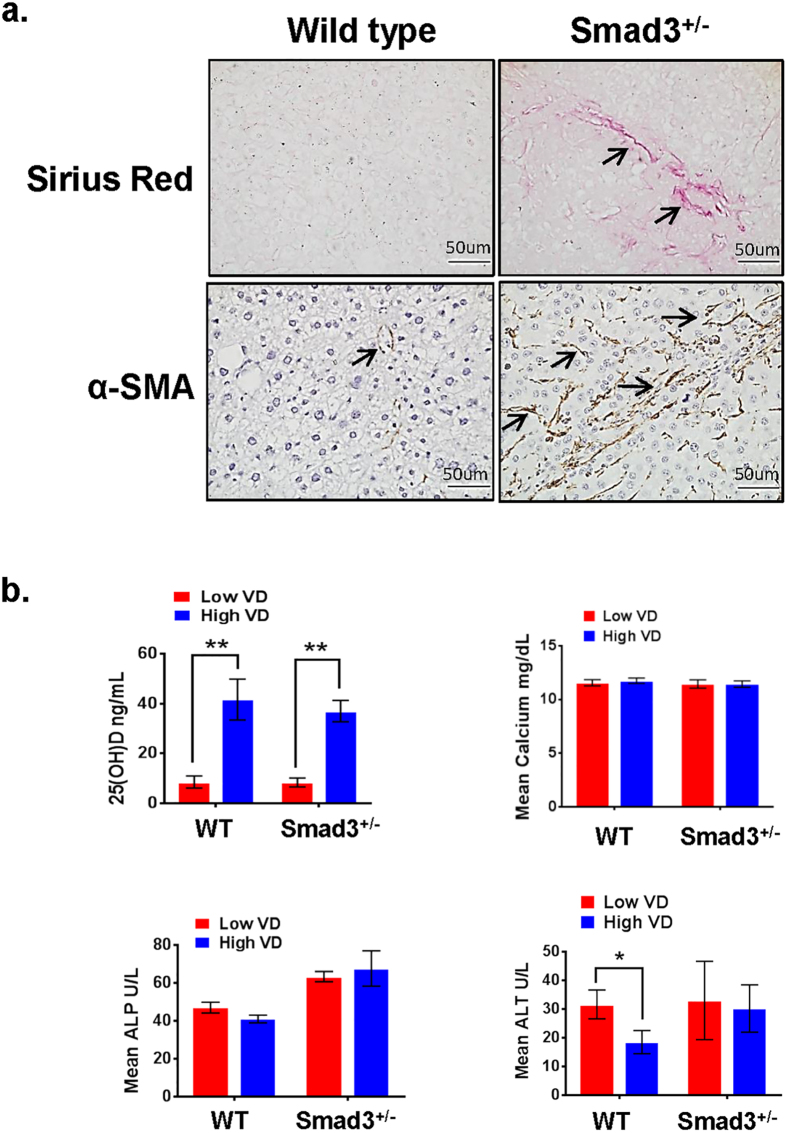
VD and Smad3 deficiency promote liver fibrosis. (**a**) Liver fibrosis in low VD treated wild type and *Smad3*^+/−^ mice was analyzed by Sirius Red and α-smooth muscle actin (α-SMA) staining. *Smad3*^+/−^ mice show higher levels of liver fibrosis. Arrows indicate fibrosis areas. (**b**) Serum VD, calcium, alanine transaminase (ALT) and alkaline phosphatase (ALP) concentrations were measured in blood from low/high VD treated wild type and *Smad3*^+/−^ mice. The serum VD levels in the VD-deficient group were lower than 20 ng/ml (deficient) VD, while the high VD treated group had VD levels higher than 30 ng/ml (normal), without any difference in calcium levels. Liver functional enzymes ALT and ALP levels were in the normal range but the ALT and ALP levels were slightly higher in the mutants than the controls. Results are presented as mean ± SD. (*p < 0.05, **p < 0.01, Student’s t-test).

In addition to increased tumor progression, *Smad3*^+/−^/VDD mice also exhibit increased liver fibrosis as indicated by Sirius red and α-smooth muscle actin (α-SMA) staining (Fig. 2a). Hepatotoxicity, VD and calcium levels in recipient mice were analyzed for serum VD, calcium, alanine transaminase (ALT) and alkaline phosphatase (ALP). Serum VD levels in the VD-deficient group were lower than 20 ng/ml (deficient) VD. The high VD treated group had VD levels higher than 30 ng/ml (normal), without any difference in calcium levels (Fig. 2b). Liver function enzymes ALT and ALP levels in all groups were in the normal range (ALT: 18.5 to 33 U/L, ALP: 41 to 67.6 U/L) (Fig. 2b).

Taken together, these results suggest that VD deficiency specifically promotes liver tumor progression under Smad3-impaired conditions.

### TLR7 is a downstream target of Smad3 and VD signaling

**Figure 3:**
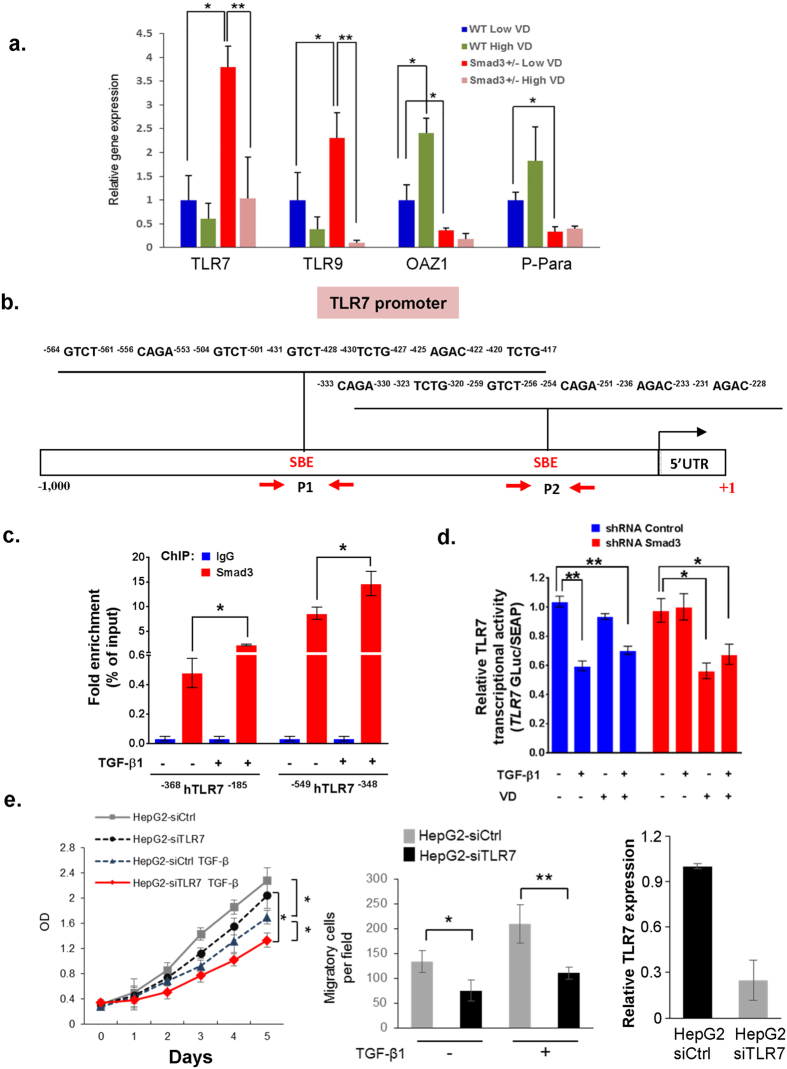
TLR7 is a downstream target of TGF-β/Smad3 and VD signaling. (**a**) Total mRNA was isolated from liver tumors of both wild type and *Smad3*^+/−^ mice which were fed either low VD or high VD regimen (n = 3 for each group). Gene expression levels of TLR7, TLR9, OAZ1, and P-Para were measured by Q-PCR. Each result shown is representative of three independent experiments. Error bars are shown as standard deviations. (*p < 0.05 [WT Low VD vs. Smad3^+/−^ Low VD], **p < 0.05 [Smad3^+/−^ Low VD vs. Smad3^+/−^ High VD], Student’s t-test). (**b**) A schematic representation of the TLR7 promoter studied by ChIP analysis. Two sets of PCR primers, designated as P1 and P2, were used for amplification of the TLR7 promoter. SBE (Smad-binding element) positions are numbered relative to the major transcription start site (+1). Black arrow indicates transcription start site. (**c**) HepG2 cells were treated with 200 pM TGF-β for 2 hrs. ChIP experiments were performed as described in Methods. (**d**) Gaussia luciferase plasmid containing a TLR7 promoter region was transfected into HepG2 shRNA-Control and HepG2 shRNA-SMAD3 cells. The secondary reporter, secreted Alkaline Phosphatase, serves as an internal control. TLR7 transcriptional activity was analyzed after 200 pM TGF-β and 100 nM VD treatment for 24 hrs. Error bars are shown as SD in (**c**,**d**). Each result shown is representative of three independent experiments (*p < 0.01, **p < 0.001, Student’s t-test). (**e**) Knockdown TLR7 suppresses HepG2 cell growth and cell migration. Cell proliferation of HepG2-siCtrl and HepG2-siTLR7 cells were assessed by colorimetric MTS assays. Transwell migration assays of HepG2-siCtrl and HepG2-siTLR7 cells were performed. Cells were treated with TGF-β1 (200 pM) for 24 hours. HepG2 cells were transfected with control-siRNA or TLR7-siRNA. The expression of TLR in HepG2-siCtrl and HepG2-siTLR7 cells were measured by Q-PCR analyses. Results are presented as mean ± SD (*p < 0.05, **p < 0.01, Student’s t-test).

Toll-like receptors are involved in hepatic inflammation and the development of hepatocellular cancer^19^. Microarray analysis demonstrates that TLR7 and TLR9 are overexpressed in human hepatocellular carcinomas (HCCs) compared with liver tissues from cirrhotic and viral hepatitis patients^20^. Chronic hepatitis patients express high levels of TLR7 and TLR4, and mice deficient in TLR4 have a marked decrease in the incidence, size, and number of chemically (DEN) induced liver cancer tumors, indicating a strong contribution of TLR signaling to hepatocarcinogenesis^21^,^22^. Moreover, TLRs have also been shown to decrease after administration of VD^23^. To elucidate the mechanistic relationship between the TLR overexpression and the increased tumor growth in mice with VD deficiency and Smad3 deletion-induced impairment of TGF-β signaling, the DEN-injected wild type and *Smad3*^+/−^ mice were fed with either high or low VD food for 4 months beginning in the 8th month of life (Fig. 1a). Liver tumors were collected and Q-PCR was performed. Our results confirm that the expression levels of TLR7, TLR9 and constituents of TGF-β signaling, OAZ1 and P-Para were significantly altered in the tumor tissues of *Smad3*^+/−^ VD-deprived mice compared to the tumor tissues of wild type VD deprived mice tumor tissues (Fig. 3a). Most importantly, TLR7 expression levels increased three-fold in *Smad3*^+/−^/VD-deprived mice compared to wild type VD-deprived mice (Fig. 3a). Moreover, high VD treatment significantly suppresses TLR7 and TRL9 expression levels in *Smad3*^+/−^ mice, and increases TGF-β signaling in wild type mice (Fig. 3a), suggesting that VD may regulate TLR7 and TLR-related genes through upregulating the TGF-β signaling pathway. Smad3 transcriptionally regulates downstream target genes via the Smad-binding element (SBE) motif, 5′-GTCT-3′^24^. We identified 22 evolutionarily conserved SBE motifs within −1,000 bp of the mouse *TLR7* promoter regions and examined Smad3 and chromatin binding activity at this *TLR7* promoter region (Fig. 3b). Examination of protein binding to the SBE motif using chromatin immunoprecipitation (ChIP) assays revealed significant enrichment of Smad3 at the promoter motif from −333 to −228 and from −564 to −417 (Fig. 3c). Importantly, Smad3 binding to *TLR7* promoter SBE motifs was increased with TGF-β treatment, suggesting that TGF-β induces Smad3 binding at the *TLR7* promoter (Fig. 3c). Luciferase reporter gene analysis revealed that the relative TLR7 transcriptional activity was reduced by TGF-β1 or TGF-β1/VD treatment in control and Smad3 knockdown cells (Fig. 3d). In Smad3 knockdown cells, the relative TLR7 promoter activity as reported via luciferase activity, decreased with VD treatment but not with TGF-β1 stimulation (Fig. 3d). Interestingly, although our Q-PCR results indicate that Smad3 inhibits TLR7 expression likely reflecting endogenous TGF-β activity (Fig. 3a), supported by the observation in cell lines, that Smad3-mediated TLR7 transcriptional regulation is TGF-β-dependent (Fig. 3d). In addition, we also find that knockdown of TLR7 significantly inhibits HepG2 cell proliferation and migration (Fig. 3e). These results suggest that VD deficiency in *Smad3*^+/−^ mutants promotes liver tumor growth through TLR7 modulation.

In summary, our mechanistic studies demonstrate that VD is involved in hepatocellular cancer possibly through TLR7 and TLR-related gene regulation.

### VD deficiency promotes hepatocellular cancer through modulating multiple genes and proteins

**Figure 4:**
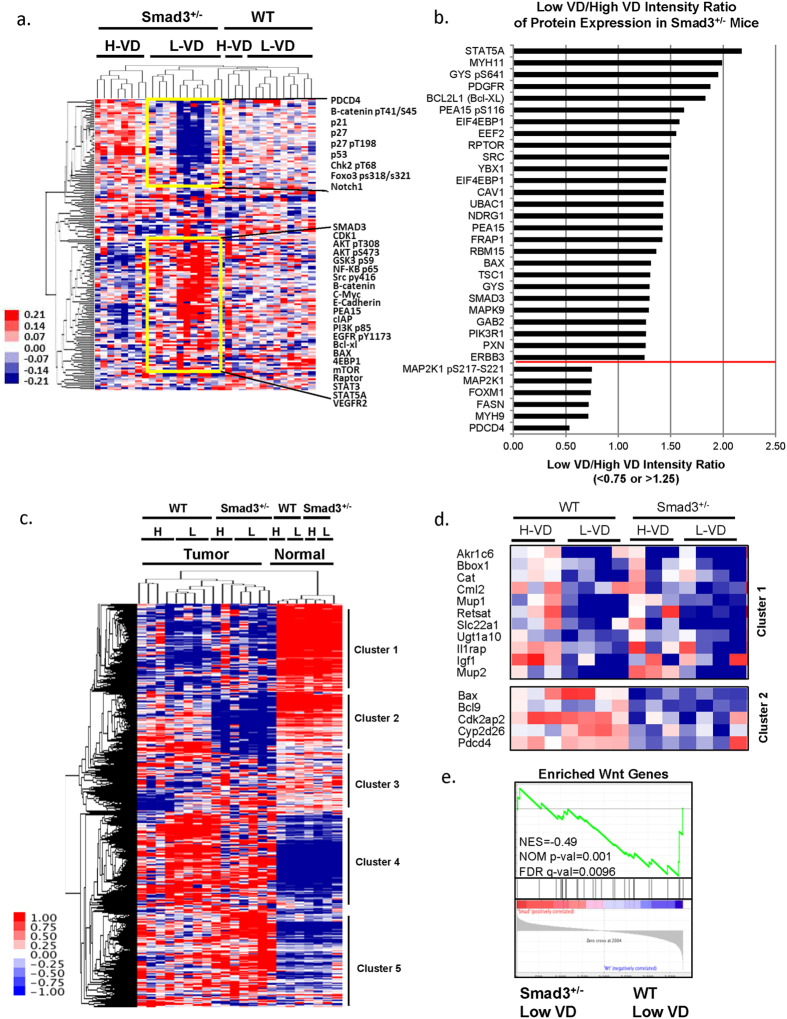
Multiple genes and proteins including WNT pathway members are altered in Smad3^+/−^ and low-VD fed mice. (**a**) Liver tumors were isolated from wild type or *Smad3*^+/−^ mice fed with either a low VD or a high VD diet. Protein lysates were evaluated with 164 antibodies for RPPA analysis. Heatmap of RPPA analysis displaying differential expression of proteins in tumor samples (cutoff: standard deviation <0.4). (**b**) Most altered proteins in *Smad3*^+/−^ mice. The ratios of low VD/high VD intensity were presented as <0.75 or >1.25 (separated by the red line). (**c**) Heatmap of the microarray results from liver tissues of wild type or *Smad3*^+/−^ mice fed with either a low VD or a high VD diet (cutoff: standard deviation <0.4; final number of probes, 4,694). (**d**) Represent genes are shown in Cluster 1 and Cluster 2. (**e**) Wnt pathway positively correlates with genes expressed in DEN-induced tumors under low VD treatment in *Smad3*^+/−^ mice. We applied GSEA to compare enriched expression of Wnt pathway genes (28 genes) in microarray gene profile in DEN-induced tumors under low VD treatment in *Smad3*^+/−^ mice and wild type mice.

To identify the most affected genes and proteins behind liver cancer formation promoted by VD deficiency, we performed reverse phase protein array (RPPA) and microarray analyses of the liver tumor tissues. RPPA results revealed that compared to the high VD-fed *Smad3*^+/−^ mice group, *Smad3*^+/−^ mice deprived of VD, showed repression of tumor suppressors such as programmed cell death 4 (PDCD4), p21, p27 and p53 and induction of oncogenic proteins such as Akt, c-Myc, mTor, Stat5A, Bcl-XL, and PEA15 (Fig. 4a). The low VD/high VD intensity ratio of protein expression is shown in Fig. 4b. The whole RPPA dataset could be found in Supplementary Dataset 1. Microarray analysis of liver tumors isolated from wild type and *Smad3*^+/−^ mice treated with high VD or low VD, displayed altered expression patterns in molecular pathways when compared with normal livers from wild type or *Smad3*^+/−^ mice treated with high VD or low VD (Fig. 4c–d and Supplementary Fig. S1). We listed the most repressed genes in cluster 1 in both low VD treated wild type and *Smad3*^+/−^ mice (Fig. 4d). Cluster 2 reveals a significant decrease in expression of genes related to VD metabolism, such as *Cyp2d26*, and antiapopotosis-related gene *Bax* and *Pdcd4* in *Smad3*^+/−^ mice fed with the VD-deficient diet (Fig. 4d). The most decreased genes in wild type mice fed with the high VD diet are shown in Supplementary Fig. S1. In contrast, high expression levels of genes were differently observed in the low VD diet in *Smad3*^+/−^ mice and the low VD diet in wild type mice (Supplementary Fig. S1). These results suggest that VD deficiency regulates liver cancer formation in a manner dependent on multiple genes and proteins. In addition, our signaling pathway analysis demonstrates that the Wnt pathway members positively correlate with the up-regulated genes in DEN-induced tumors under low VD treatment in *Smad3*^+/−^ mice (Fig. 4e). The whole microarray dataset could be found in Supplementary Dataset 2. The gene list used in Fig. 4e could be found in Supplementary Dataset 3.

**Figure 5:**
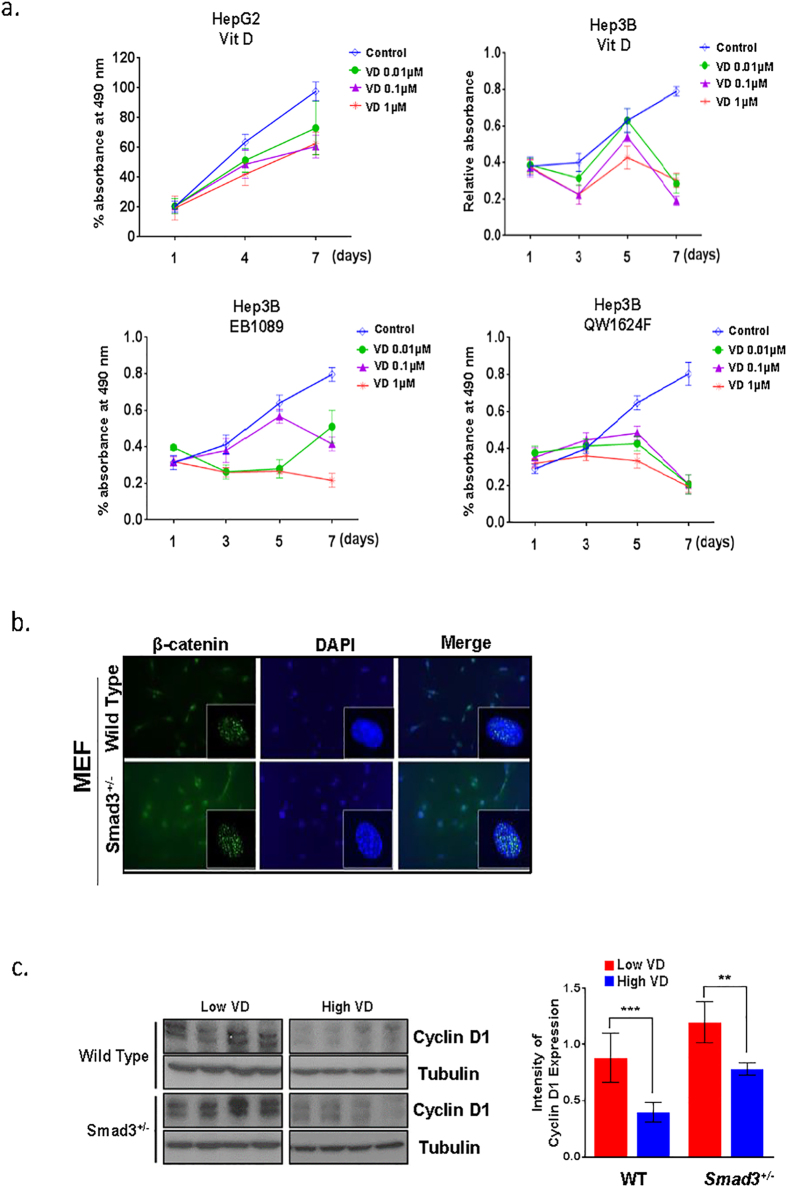
VDR signaling pathway reduces the proliferation rate of liver cancer cells, and loss of Smad3 increased levels of nuclear β-catenin. (**a**) HepG2 and Hep3B cells were treated 10 nM, 100 nM or 1000 nM of VD, EB1089, or QW1624F and with control (100% ethanol), and this treatment continued for 7 days. Viability of the cells was assessed on indicated day using MTS reagent (Promega). (**b**) β-catenin nuclear localization intensity was measured by immunofluorescence staining with phospho-β-catenin antibodies and DAPI in control and *Smad3*^+/−^ MEF cells. (**c**) Western blot analysis of *cyclin D1* in liver tissue taken from wild type and *Smad3*^+/−^ mice fed with either high or low VD (4 mice in each group). Intensity of cyclin D1 expression in each group of mice was displayed in bar graph (right panel). (**P < 0.001, ***P < 0.0005, Student’s t-test).

Calcitriol (1,25(OH)_2_D_3_) binds to VDR and takes part in both genomic and nongenomic pathways that regulate VDR target gene expression^12^,^25^. One of the important non-calcemic effects of calcitriol is the anti-proliferative effect which may help in preventing and treating cancer. To determine whether the VDR pathway affects *in vitro* proliferation rates of liver cancer cell lines, HepG2 and Hep3B human liver cancer cell lines were treated with increasing doses of VD and VD-analogs, EB1089 and QW1624F (0.01–1 μM), for 7 days (Fig. 5a). All VD and VD-analogs inhibited proliferation of liver cancer cell lines after 7 days of treatment. This result demonstrates that the VDR signaling pathway reduces the proliferation rate of liver cancer cells.

Human liver cancer is often characterized by alterations in the cadherin/catenin adhesion and signaling system, either as a result of activating mutations in β-catenin or inactivation of the E-cadherin. The TGF-β/Smad pathway can also interact with the β-catenin/T-cell factor (TCF) transcriptional machinery and co-operatively regulate transcription of a number of genes. Moreover, β-catenin can bind directly to VDR and potentiate its transactivation and differentiation activity^26^. The pathway was significantly affected in our Smad3 mutants deprived of VD (Fig. 4e). Consistent with our findings, we also detected increased levels of nuclear β-catenin in *Smad3*^+/−^ MEF cells, Smad3 knock down and VDR knock down in HepG2 cells (Fig. 5b, Supplementary Fig. S2). Furthermore, expression levels of β-catenin target gene, *cyclin D1*, are increased in *Smad3*^+/−^ mice (Fig. 5c). In summary, disruption of Smad3 leads to aberrant activation of Wnt signaling, further supporting our conclusions derived from GSEA analysis in Fig. 4e.

### High-dose VD treatment induced TGF-β signaling proteins in cirrhosis and liver cancer patients

**Figure 6:**
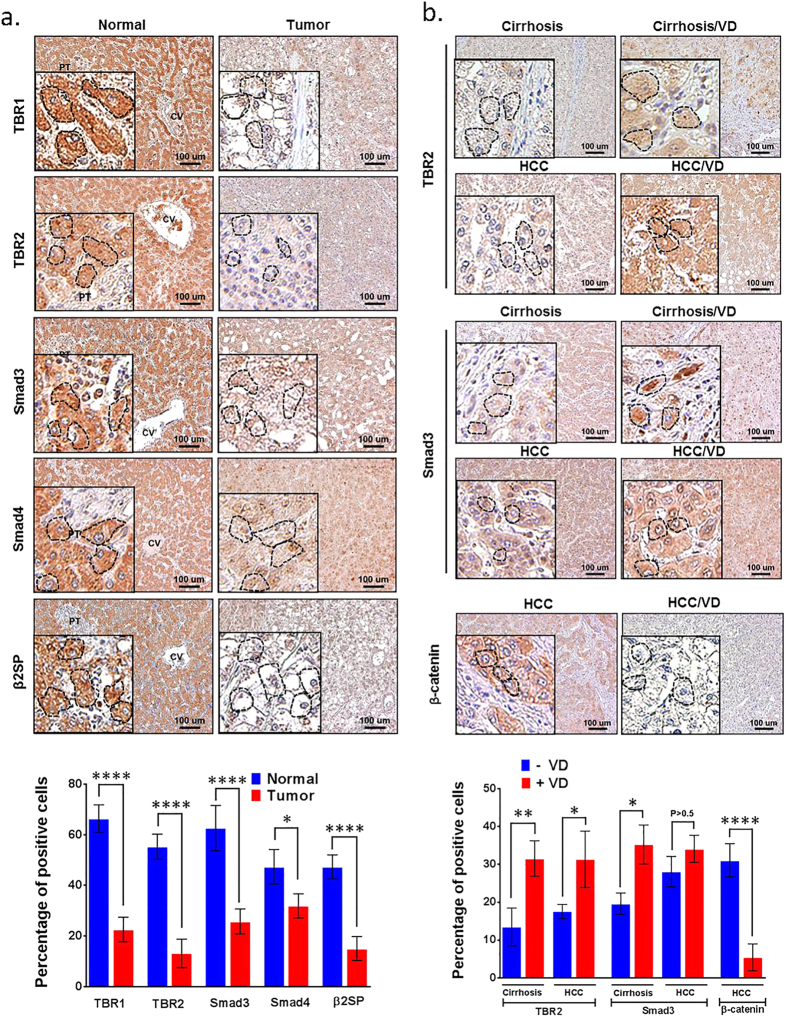
High-dose VD treatment induces Smad3, β2SP and TBR2 expression while suppressing β-catenin in the liver of cirrhotic or hepatocellular cancer patients. (**a**) Patients were treated with or without VD (50,000 IU / week) for 26 weeks. IHC staining for TBR1, TBR2, β2SP, Smad3, and Smad4 were performed in human tissue specimens of normal liver (n = 12) and HCC (n = 11). Quantification of IHC staining is shown in the bar graph (lower panel). (**b**) IHC staining for TBR2, Smad3 and β-catenin were performed in human liver tissue specimens of cirrhosis (n = 10), cirrhosis with VD-treatment (n = 7), HCC (n = 11), and HCC with VD-treatment (n = 12). Quantification of IHC staining is shown in the bar graph (lower panel). Magnification ×20; the insets magnification ×60. Scale bar, 100 μm. Circles indicate representative hepatocytes. Error bars are shown as standard deviations. (*P < 0.01, **P < 0.001, ****P < 0.0001, Student’s t-test).

Because loss of TGF-β signaling proteins (Smad3, β2SP) has been implicated in liver cancer in mice^27^, we examined the expression levels of TGF-β signaling proteins in human livers. All human tissue procedures were approved by the Institutional Review Board of University of Texas M.D. Anderson Cancer Center, Houston. Immunohistochemistry (IHC) staining analysis revealed that TBR1, TBR2, Smad3, Smad4 and β2SP protein expression levels were significantly lower in liver tumors (n = 11) compared to normal livers (n = 12) (Fig. 6a). Quantification of IHC labeling is shown in the bar graph (Fig. 6a). Two groups of patients (10 patients with cirrhosis and 11 patients with hepatocellular cancer in group 1; 7 patients with cirrhosis and 12 patients with hepatocellular cancer in group 2) were treated with either a placebo or a high dose of VD for 4 months. Liver tissues were collected both before and after treatment. IHC staining showed that TBR2 and Smad3 expression were restored by VD treatment in both liver tissues from cirrhosis and HCC patients, whereas β-catenin expression was repressed by VD treatment in liver tissues from HCC patients (Fig. 6b). Quantification of IHC labeling is shown in the bar graphs (Fig. 6b). These results implicate loss of TGF-β signaling proteins in human hepatocellular cancer and cirrhosis, and suggest that VD may serve as a candidate treatment reagent to restore the lost TGF-β signaling in these patients.

### Somatic mutations and mRNA alterations of VD-related genes are correlated with the TGF-β signaling pathway

**Figure 7:**
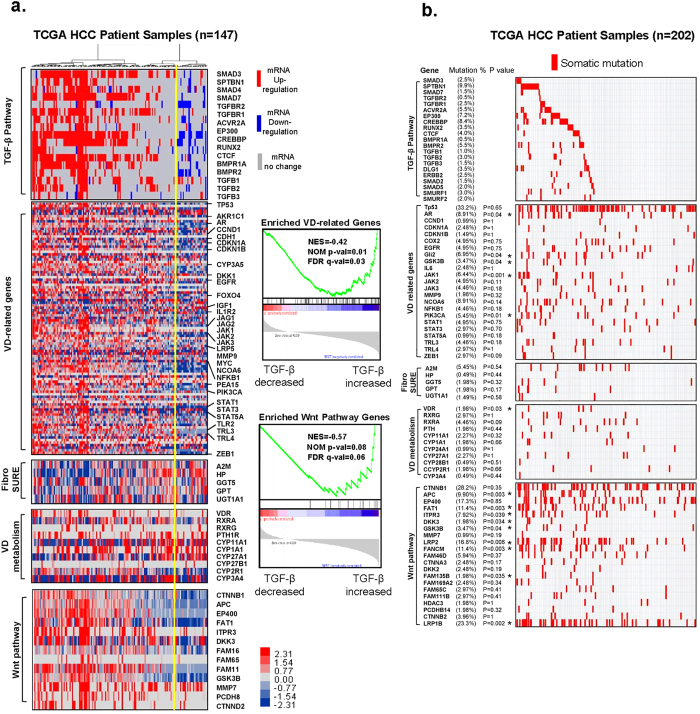
VD-related genes and hepatic fibrosis genes are highly mutated and correlate with TGF-β signaling transduction pathways in hepatocellular carcinomas. (**a**) Analysis of tumor transcriptomes reveals frequent abnormalities in the TGF-β signaling pathway in hepatocellular cancer patients. Data for 147 cases reported in TCGA were downloaded from cBioPortal. VD-related genes, FibroSURE, VD metabolism, and the Wnt pathway were analyzed in parallel. The genomics selection criterion was the mRNA expression z-score ± 2.0 standard deviations. Changes in frequency are indicated as percentages of all cases. Upregulation is indicated in red; downregulation is indicated in blue. We applied GSEA to compare enriched expression of VD related genes and Wnt pathway genes with TGF-β signatures (right panel). (**b**) Landscape of somatic mutations in the TGF-β pathway genes in 202 TCGA HCC samples. The percentages of samples with mutations in a given gene are shown. p-values were computed using Fisher’s Exact test of independence.

In order to gain insight into the genetic alterations affecting members of the TGF-β superfamily, we performed an unbiased analysis of 147 cases reported in The Cancer Genome Atlas (TCGA) database. Based on the mRNA alterations in the heat map and the dendrogram based on hierarchical clustering, the TGF-β superfamily gene clusters revealed two distinct TGF-β signatures: a blue signature for reduced mRNA levels of TGF-β superfamily genes and a red signature for increased mRNA levels of TGF-β–related genes (Fig. 7a, left panel). We then determined whether there were any correlations between the dysregulated TGF-β superfamily pathway and the VD-related pathway, FibroSURE, VD metabolism, and Wnt pathway genes by applying GSEA to compare enriched expression of VD-related genes and Wnt pathway genes against these signatures. Down-regulation of TGF-β superfamily genes significantly correlated with decreased levels of VD-related genes and Wnt pathway genes, suggesting that loss of TGF-β superfamily genes is significantly associated with loss of VD-related genes and Wnt pathway genes (Fig. 7a, right panel). The gene lists of each signal pathway summarized several Molecular Signatures Database (MSigDB) from GSEA gene sets and can be found in Supplementary Dataset 4.

For somatic mutations in genes involved in the TGF-β signaling pathway, VD-related genes, FibroSure, VD metabolism and the Wnt pathway, we analyzed 202 hepatocellular cancer cases reported in the TCGA (Fig. 7b). Genes that were highly mutated are as follows: in the TGF-β signaling pathway, *SPTBN1 (β2SP), Smad3, Tgfb1* and *Tgfbr1*; in the VD-related gene group, *Ccnd1, Egfr, Il-6, Jak1, Stat5a* and *Tlr4*; in the FibroSure group, *A2m, Hp, Ggt5, Gpt* and *Ugt1a1*; in the VD metabolism group, *VDR, Rxrg*, and *Cyp27a1*; in the Wnt pathway, *Ctnnb1* and *LRP2* (Fig. 7b). The full gene list along with corresponding data could be found in Supplementary Dataset 5. We found that several gene mutations in these categorized groups correlated to the mutations in TGF-β pathway genes.

Taken together, our TCGA analysis reveals that alteration of TGF-β signaling strongly correlates with alteration of VD-related genes and the Wnt signaling pathway.

## Discussion

In this study, we examined the effect of Vitamin D in the context of TGF-β signaling pathway disruption utilizing *Smad3*^+/−^ and wild type mice. Specifically, we observed that the tumor burden increased over three-fold and the liver-to-body weight ratio was significantly higher in *Smad3*^+/−^ mice deprived of VD compared to *Smad3*^+/−^ animals treated with high-dose VD. Cell proliferation assays further support increased liver tumor progression in VD-deprived *Smad3*^+/−^ mice. A low VD regimen promotes tumor growth in the context of TGF-β/Smad3 disruption. Since we did not observe a comparable effect in wild type mice, the results suggest that VD tumor suppression is modulated by aberrant Smad3/TGF-β signaling. In addition to tumor growth, *Smad3*^+/−^ VD-deficient mice also exhibit increased liver fibrosis compared to controls, reflective of epithelial-mesenchymal transition (EMT) in liver tissues in the mutant mice. The deprivation of Vitamin D with underlying chronic inflammation (the latter being well described) in the mutant mice therefore appears to lead to this unexpected phenotype^28^.

In all mouse models, high VD intervention 8 months after DEN treatment via intraperitoneal injection did not affect the total number of tumors. Nor did we observe any alterations in lung adenocarcinomas in any of the DEN treated groups, suggesting the liver specificity of our findings. Microarray and RPPA analyses of tumors from high VD or VD-deficient food fed mice showed that expression patterns of tumor suppressors such as programmed cell death 4 (PDCD4), p21, p27 and p53, oncogenic proteins such as Akt, c-Myc, mTor, Stat5A, Bcl-XL, PEA15, and genes related to VD metabolism, such as *Cyp2d26*, were significantly altered in the *Smad3*^+/−^/VD deficient group. These findings suggest that Smad3-mediated TGF-β signaling plays an important role in suppression of liver tumor formation in the context of VD deficiency, and that replacing with high dose VD at later stages in life may not be efficacious in rescuing the defects.

Liver disease is also characterized by increased production of inflammatory cytokines, including TNF-α and IL-6^29^. In addition, the expression levels of TLR family members, which are key proteins in innate immune system, are also altered. High expression levels of TLR7 and TLR9 have been observed in liver tissue microarrays of patients with cirrhosis, viral hepatitis, and hepatocellular cancer^20^. VD or its metabolites are known to suppress pro-inflammatory cytokines such as TNF-α and IL-6 expression^30^,^31^,^32^,^33^. Interestingly, TLR7 expression levels have been observed to negatively correlate with VD levels in patients with hepatocellular cancers^23^. Our cellular studies revealed that TLR7 transcriptional activity is directly regulated by Smad3 and VD. Furthermore, we also identified two Smad3 binding sites at the TLR7 promoter region. VDR is highly expressed in hepatic stellate cells (HSC), indicative of its role in regulating liver fibrosis. VDR has been demonstrated to behave as an antagonist to Smad3 and prevent liver fibrosis in stellate cells^10^. In this study, we reveal that VD also has an *in vivo* anti-inflammatory epithelial effect on liver tumor development. That VD inhibits inflammation through suppressing TLR7 expression only in the background of Smad3 deficiency fits well with the model that VDR and Smad3 response elements are co-localized within one nucleosomal window^10^. Moreover, we also demonstrate that TLR7 silencing in HepG2 cells significantly inhibits cell proliferation and migration. Therefore, our studies confirm and identify a new mechanism for the VD anti-inflammatory effect through suppression of TLR7 expression in the background of Smad3 deficiency.

Multiple observations indicate that VD deficiency is highly prevalent in cirrhotic patients. Thus, there is already a strong rationale for prescribing VD supplements for patients with advanced liver disease to prevent metabolic bone disease^8^,^9^. In a retrospective study from our center examining the prevalence of VD deficiency in 330 patients with cirrhosis, we found that the overall prevalence of VD deficiency was 93%, with severe VD deficiency (defined as 25(OH)D level < 7 ng/ml) noted in 28% of patients (unpublished data). These findings indicate that, regardless of cirrhosis etiology, VD deficiency occurs almost universally in patients with cirrhosis, and supplemental VD treatment appears justified. However, epidemiological data linking VD deficiency and liver cancer development are contradictory. A case-control study in China showed that the incidence of liver cancer in chronic liver disease appears to negate any significant association between liver cancer risk and circulating levels of VD^34^. In contrast, a recent sub-analysis of the European Prospective Investigation into Cancer and Nutrition (EPIC) showed that in individuals with VD deficiency, the likelihood of developing liver cancer was 1.82 times higher than the likelihood in individuals without VD deficiency^35^. These conflicting reports highlight the inherent inconsistencies of epidemiological data, and hence the need for combined mechanistic and clinical studies to elucidate the role of VD in liver carcinogenesis.

TGF-β has a complex role in liver disease development, in which it induces inflammation and fibrosis as well as apoptosis with tumor suppression, and later hepatocyte proliferation that drives liver cancer progression^36^,^37^. Moreover, patients with activated TGF-β signaling develop liver cancer in the background of significant hepatic fibrosis^37^. In agreement with these findings, we observed higher expression of TGF-β signaling proteins in normal human liver than in liver tumors. We further observed higher β-catenin nuclear levels under *Smad3*^+/−^ conditions, with increased levels of β-catenin target gene expression, including *Cyclin D1*. Further analysis of VD treatment in human cirrhosis and hepatocellular cancer revealed that VD treatment restored expression levels of TGF-β pathway members and destabilized β-catenin, suggesting that patients with an inactivated TGF-β signature could potentially benefit from VD treatment.

Lastly, we analyzed somatic mutations and alterations in genes involved in VD metabolism, VD-related genes, and the TGF-β superfamily utilizing the TCGA database of 147 patients with liver cancer. The whole genome and transcriptomic analyses reveal that somatic mutations in genes involved in VD metabolism and VD-related genes occur frequently in hepatocellular cancers correlating with the TGF-β superfamily, suggesting that combined inactivating somatic mutations in VD-related genes and the TGF-β signaling members play a critical role in liver tumorigenesis.

In summary, our findings strongly suggest that VD treatment has the potential to play a significant role in prevention of liver cancer progression in the context of inactivation of TGF-β signaling in patient populations with underlying VD deficiency.

## Methods

### Mice and Diet

Wild type and *Smad3*^+/−^ male mice were housed in a specific pathogen-free facility. Mice were treated with 50 mg/kg of the liver carcinogen diethylnitrosamine via intraperitoneal injection at 14 days of age. At 8 months, the diet was changed from regular chow (2,200 IU VD/kg) to one of two diets: lower than normal VD (200 IU VD/kg) to replicate the deficiency found in patients, or high VD (10,000 IU VD/kg). All diets were manufactured by PMI Nutrition International, St. Louis, MO (LabDiet/TestDiet), based on the AIN-93M diet. The mice were euthanized at 1 year of age, and the livers were harvested for tissue analysis. All experiments were approved by the Institutional Animal Care and Use Committee of MD Anderson Cancer Center, and all methods were carried out in accordance with the approved guidelines.

### Quantitative Real-Time PCR Analysis

Total RNA was extracted using TRIzol reagent according to the manufacturer’s instructions. Reverse transcription was performed using a SuperScript III First-Strand Kit (Invitrogen, Carlsbad, CA). Each complementary DNA (10 ng) was amplified in triplicate with an iQ SYBR Green Supermix PCR Kit (Bio-Rad, Hercules, CA) for 40 cycles. The primers used were: TLR7-mouse-F: ATGTGGACACGGAAGAGACAA; TLR7-mouse-R: ACCATCGAAACCCAAAGACTC; TLR9-mouse-F: ATGGTTCTCCGTCGAAGGACT; TLR9-mouse-R: GAGGCTTCAGCTCACAGGG; OAZ1-mouse-F: CGCACCATGCCGCTTCTTA; OAZ1-mouse-R: ATCCCGCTGACTGTTCCCT; PPARA-mouse-F: AACATCGAGTGTCGAATATGTGG; PPARA-mouse-r: CCGAATAGTTCGCCGAAAGAA; TLR7- human -F: TCCTTGGGGCTAGATGGTTTC; TLR7- human -R: TCCACGATCACATGGTTCTTTG.

### Cell Culture

Cell lines HepG2 and Hep3B were cultured in DMEM supplemented with 10% fetal bovine serum. Lentiviral particles containing shRNA to Smad3 (sc-38376), control shRNA (sc-108080), siRNA to TLR7 (sc-40266) or control siRNA (sc-44231) were purchased from Santa Cruz Biotechnology and were used to infect HepG2 cells. siRNA for β-catenin (L-003448-00-0005) and siRNA control Non-targeting Pool (# D-001810-10-05) were purchased from Dharmacon (Pittsburgh PA).

### Chromatin immunoprecipitation (ChIP) Assays

ChIP assays were performed using EZ ChIP assay kit (# P-2002, Millipore, Billerica, MA, USA), following the manufacturer’s instructions. HepG2 cells were washed, cross-linked with formaldehyde (1% final concentration), and sonicated on ice to fragment the chromatin into an average length of 500 bp to 1 kb. The lysates were diluted using chromatin dilution buffer. Smad3 antibody (Cell Signaling, Danvers, MA) was used to immunoprecipitate the respective antigens at 4 °C overnight. For each ChIP, a fraction of the input chromatin (10%) was also processed for DNA purification; a mock immunoprecipitation with unrelated IgG (Cell Signaling, Danvers, MA) antiserum was carried out in parallel. With the DNA isolated at the end of the ChIP analysis, quantitative PCR was performed using the TLR7 promoter-specific primers. Two PCR primer sets were used for the ChIP assay; Primer 1F: 5′- AGAGCAAGGGTGCTACCAAA-3′ (from −185 to −204) R: 5′-AAGCACGGGGTTCCTCTTAT-3′ (from −349 to −368), Primer 2 F: 5′- TCCTTTCCAGCTGGGTCTAA-3′ (from −348 to −368) R: 5′-TTTGGTAGCACCCTTGCTCT- 3′ (from −530 to −549).

### Luciferase reporter assays

HepG2 cells were transfected with indicated plasmids using Lipofectamine 2000 (Invitrogen, Carlsbad, CA) according to the manufacturer’s manuals. The reporter clone containing *TLR7* 5′-flanking sequence cloned in front of the secreted Gaussia luciferase gene and secreted alkaline phosphatase (SEAP) genes was obtained from Genecopoeia (Rockville, MD). Secreted Gaussia luciferase assay were performed using the Secreted-Pair dual luminescence assay kit (Genecopoeia) according to manufacturer’s manuals. Luciferase activity was normalized to Secreted SEAP assay signal.

### Reverse phase protein array (RPPA)

Protein lysate was prepared from each mouse tumor tissue. Samples were applied for microcentrifugation at 14,000 rpm for 10 minutes followed by adding lysis buffer. Supernatants were collected and protein concentration was measured using the BCA Reaction Kit (Pierce Biotechnology, Inc., Rockford, IL). Equal amount of lysate was mixed with SDS sample buffer without bromophenol blue [5× SDS sample buffer, which contained 50% glycerol, 10% SDS, and 0.31 mol/L Tris-HCl (pH 6.8)]. Tumor lysates were two-fold-serial diluted for 5 dilutions (from undiluted to 1:16 dilution) and arrayed on a nitrocellulose-coated slide in 11 × 11 formats. Samples were probed with antibodies by CSA amplification approach and visualized by DAB colorimetric reaction. Slides were scanned on a flatbed scanner to produce 16-bit tiff images. Spots from tiff images were identified and the density was quantified by Array-Pro Analyzer.

### RPPA Data Processing and Statistical Analysis

Relative protein levels were determined by interpolation of each dilution curve from the “standard curve” (supercurve) of the slide (antibody). Supercurve is constructed by a script in R written by Bioinformatics. These values (Log2 values) are defined as Supercurve Log2 (Raw) value. All the data points were normalized for protein loading and transformed to linear value designated as “Linear after normalization”. “Linear after normalization” value was transformed to Log2 value, and then median-centered for Hierarchical Cluster analysis.

### Microarray Analysis

Total RNA was extracted from the indicated samples using a mirVana RNA Isolation Labeling Kit (Ambion, Inc., Life Technologies, Grand Island, NY). 500 ng of total RNA were used for labeling and hybridization according to the manufacturer’s protocols (Illumina Inc., San Diego, CA). After the bead chips (Sentrix Human v.3 HT-12) were scanned with an Illumina BeadArray Reader, the microarray data were normalized using the quantile normalization method in the Linear Models for Microarray Data (LIMMA) package in the R language environment. The expression level of each gene was transformed into a logarithm 2-base before additional analysis was performed. Gene expression was used for hierarchical clustering after performing gene median centering and selecting genes with over 1 standard deviation across samples. Data are presented in matrix format in which rows represent the expression level of gene feature in an individual tissue and columns represent each tissue. The red and blue colors in cells reflect relative high and low expression levels respectively. Filtering process was performed by genes centered by median value, filtered by variance of expression (cut-off: SD 0.4).

### Serum VD and Calcium Levels and Tissue Collection

After the mice were euthanized, blood was obtained via cardiac puncture. Serum samples were submitted to Heartland Assays (Ames, IA) for quantification of 25-hydroxy-VD (radioimmunoassay), ALT, ALP, and calcium levels. Liver and lung tissues were fixed in 10% phosphate-buffered formalin and processed for routine histologic examination. For RPPA or microarray study, liver tumors were rinsed gently in sterile PBS to remove bloody material and stored at −80 °C until protein or DNA extraction.

### Proliferation assays

1 × 10^3^ HepG2 and Hep3B cells were plated in a 96-well plate. The next day, the cells were treated with vehicle (100% ethanol), 10 nM, 100 nM or 1000 nM of VD, EB1089, or QW1624F and this treatment continued for 7 days. Medium was replaced and reagents were added every 72 hours. Viability of the cells was assessed on indicated day using MTS reagent (Promega, Fitchburg, WI).

### Immunoblotting

Cells were lysed with lysis buffer [20 mM Tris, 100 mM NaCl, 0.5% Nonidet P-40, 0.5% Triton X-100, 1 mM EDTA]. Fresh protease/phosphatase inhibitors [5 mM NaV, 1 mM NaF, 1 μM DTT, 0.1 mg/mL pepstatin A, 1 mM PMSF, and 1,000× Complete Mixture Protease Inhibitors] were added to the lysis buffer. Protein lysates were standardized, and equal amounts of proteins were subjected to immunoblot analysis.

### Immunohistochemistry (IHC)

4 μm sections were de-paraffinized in two changes of xylenes, rehydrated sequentially in ethanol, washed in IHC wash buffer [0.5% Triton X-100 in PBS], and rinsed in water. Slides were submerged in buffer A [10 mM sodium citrate pH 6.0], and antigen unmasking was performed in the Retriever 2100 (Aptum) according to manufacturer’s protocol. The slides were allowed to cool, washed three times, incubated with 3% H_2_O_2_ to block endogenous peroxidase activity, washed three times, and blocked with 10% normal horse serum in IHC wash buffer for one hour. Slides were incubated with primary antibodies for α-SMA antibodies (1:500, ABT1487, EMD Millipore, Billerica, MT) overnight at 4 °C. The next day, slides were washed three times, and incubated with 1:500 biotinylated goat anti-rabbit secondary antibody for one hour at room temperature. Specimens were washed three times, treated with ABC reagent (Vector Labs, Burlingame, CA) for 30 minutes, followed by three washes. Finally, slides were stained with the DAB (Diaminebenzidine) substrate kit (Vector Labs), inactivated in water, counterstained with hematoxylin, dehydrated sequentially with ethanol, then cleared in two changes of xylenes, and mounted in DPX mounting media. Immunohistochemical staining for the human samples was performed using the Histostain-Plus Kit from Zymed/Invitrogen (Carlsbad, CA) according to the manufacturer’s instructions. Antibodies used were TBR2, β2SP and β-catenin (1:200, sc-220, Santa Cruz Biotechnology, Dallas, TX; 1:175, custom antibody; 1:50, 05-665, Upstate Biotechnology, Lake Placid, NY, respectively).

### Human Genomics

We analyzed the transcriptome of 147 hepatocellular cancers and screened for mutations in 202 HCCs from The Cancer Genome Atlas (TCGA). We first performed hierarchical clustering using the TCGA whole-transcriptome RNA sequencing database of 147 HCC cases (downloaded on November 2013). The fragments per kilobase of exon per million fragments mapped (FPKM) values of 18 TGF-β superfamily genes in TCGA HCC patient samples were first transformed to a ternary scale (−1, 0, 1). If the FPKM value of a gene in a patient sample fell below the 2.5% quartile of the reference population (50 normal adjacent tissues or all tumors that are diploid for the gene of interest), then the FPKM value was transformed to −1. If the FPKM value of a gene in a patient sample fell above the 97.5% quartile of the reference population, then the FPKM value was transformed to 1. Any FPKM values between 2.5% and 97.5 quartile were transformed to 0.

### GSEA Analysis

GSEA was performed by using GSEA software with the metric for ranking genes as “signal-to-noise” and the enrichment statistic as “weighted”. GSEA for Fig. 7a was performed using TCGA dataset downloaded from cBioportal tools^38^,^39^. GSEA results were considered significant when the nominal (NOM) p value was less than 0.05 and false discovery rate (FDR) q-value was less than 0.25.

### Sirius Red Staining

Sirius Red staining was performed using a Sirius Red/Fast Green Collagen Staining Kit (Chondrex, Inc), following the manufacturer’s protocol. Labeled slides were mounted with DPX mounting media.

## Additional Information

**How to cite this article**: Chen, J. *et al*. Vitamin D Deficiency Promotes Liver Tumor Growth in Transforming Growth Factor-β/Smad3-Deficient Mice Through Wnt and Toll-like Receptor 7 Pathway Modulation. *Sci. Rep.* **6**, 30217; doi: 10.1038/srep30217 (2016).

## Supplementary Material

Supplementary Information

Supplementary Dataset 1

Supplementary Dataset 2

Supplementary Dataset 3

Supplementary Dataset 4

Supplementary Dataset 5
