# Supplementary material for: Vitamin D Deficiency Promotes Liver Tumor Growth in Transforming Growth Factor-β/Smad3-Deficient Mice Through Wnt and Toll-like Receptor 7 Pathway Modulation
Source: Sci Rep. 2016 Jul 26;6:30217. doi: 10.1038/srep30217 (PMC4960540; doi:10.1038/srep30217)
Supplement: Supplementary Information [file srep30217-s1.pdf]

Vitamin D Deficiency Promotes Liver Tumor Growth in Transforming Growth Factor-β/Smad3-Deficient Mice through Wnt and Toll-like Receptor 7 Pathway Modulation

Jian Chen, Lior H. Katz, Nina M. Muñoz, Shoujun Gu, Ji-hyun Shin, Wilma S. Jogunoori, Mi-Hye Lee, Mitchell D. Belkin, Sang-Bae Kim, Jon C White, Jaclyn Andricovich, Alexandros Tzatsos, Shulin Li, Sang Soo Kim, Kirti Shetty, Bibhuti Mishra, Asif Rashid, Ju-Seog Lee and Lopa Mishra*

Supplementary Figure S1. Multiple genes are altered in *Smad3*$^{+/-}$ and low-VD fed mice. Heatmap showing microarray results from liver tissue of wild-type or *Smad3*$^{+/-}$ mice fed with either a low-VD or a high-VD diet (cutoff: standard deviation < 0.4; final number of probes, 4,694). Represent genes were shown in Cluster 3, 4 and 5.

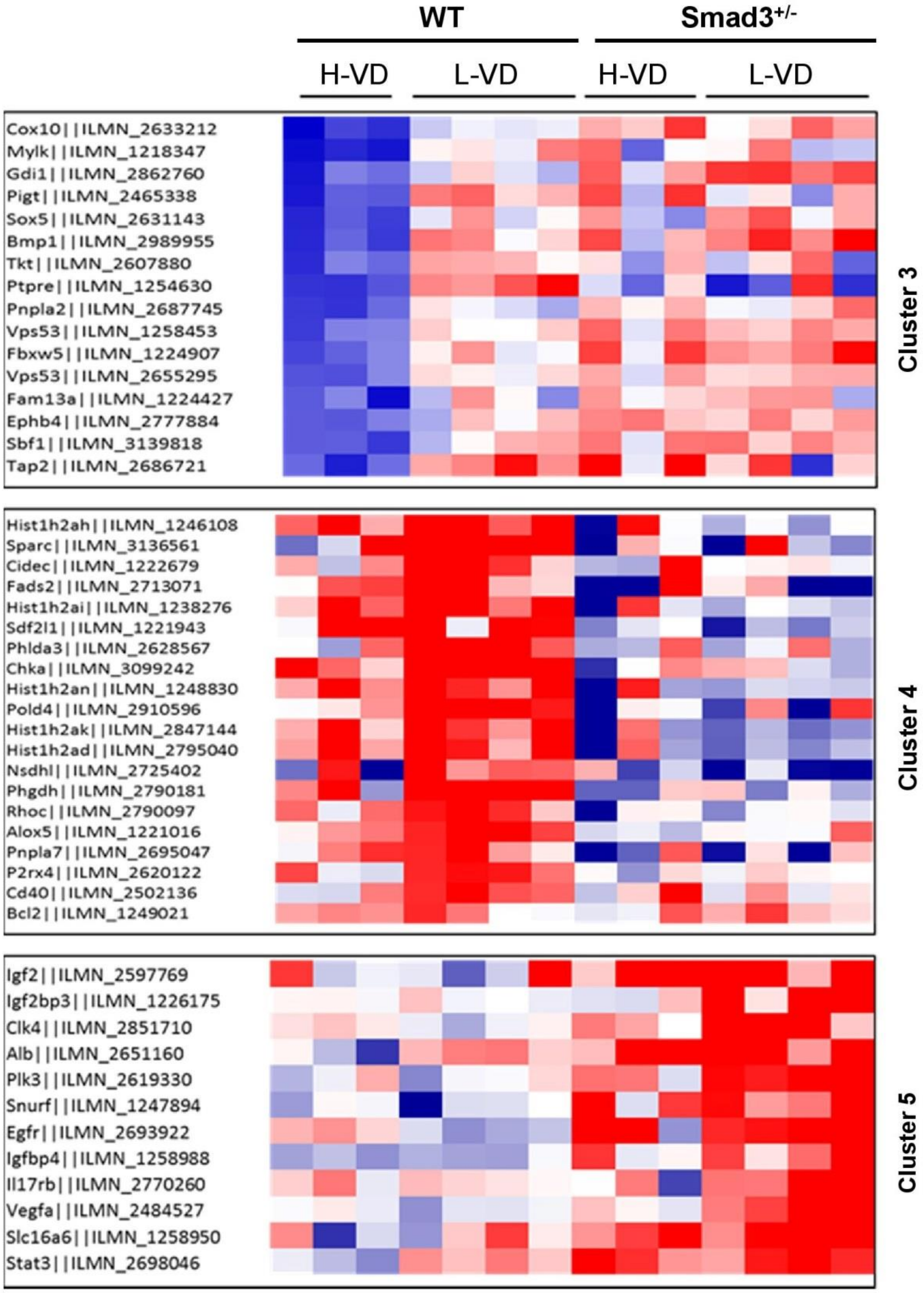

Supplementary Figure S2. Loss of VDR/Smad3 increased levels of nuclear β-catenin. β-catenin nuclear localization intensity was measured by immunofluorescence staining with phospho-β-catenin antibodies and DAPI. HepG2 cells were transfected with lentiviral shRNA for Smad3 (a), or transfected with siRNA for VDR (b). The nuclear localization intensity of β-catenin was measured with MetaMorph image analysis software. Bar graph presents mean staining intensities. (** P<0.001, Student's t-test.)

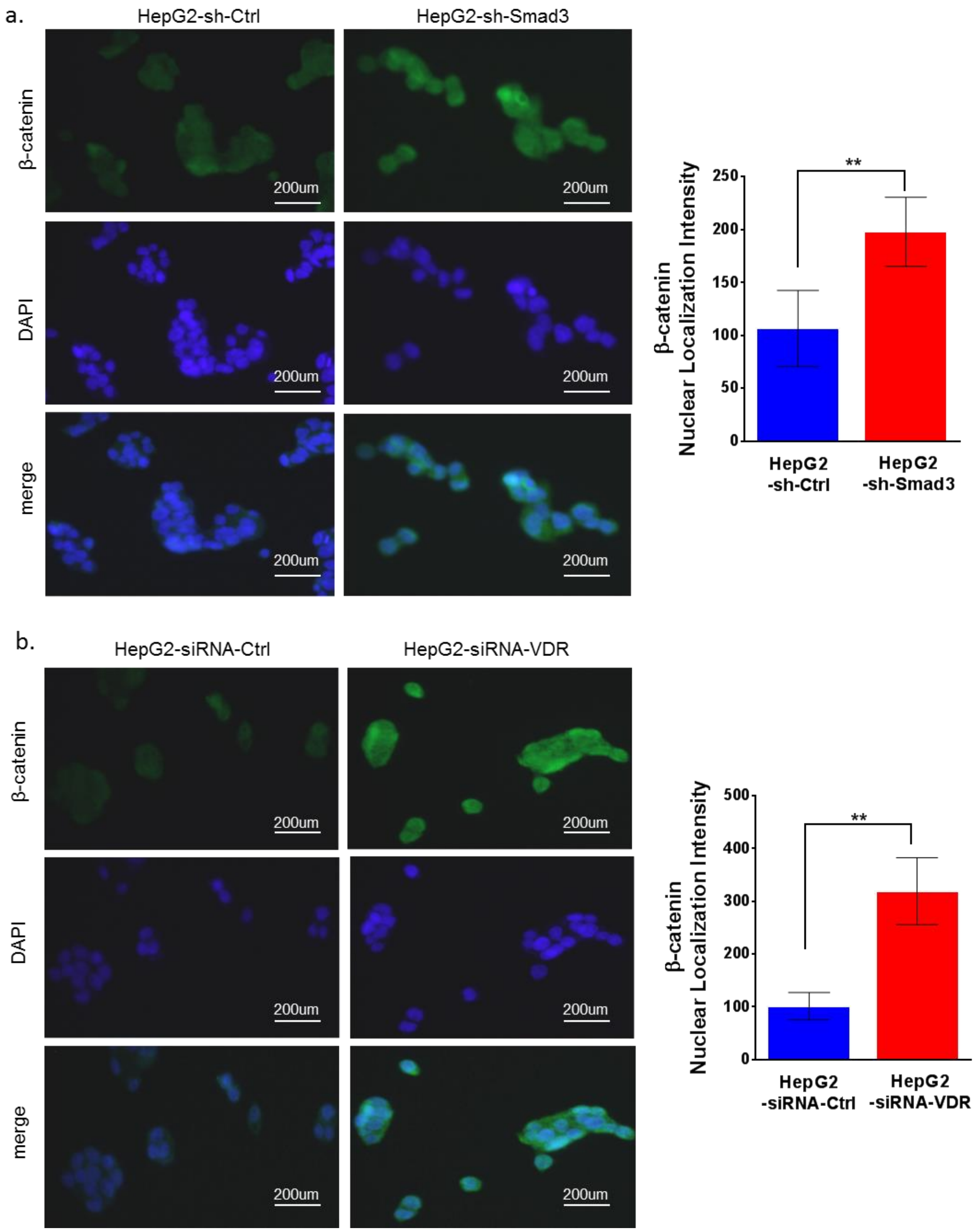

Supplementary Table S1. Tables of liver-to-body weight ratio, liver tumor number and lung tumor number.

Liver Tumor Number

	**P value**	**Low VD**	**High VD**	**Difference**
Wild type	0.078	11.0	5.0	6.0
Smad3$^{+/-}$	0.605	7.4	6.1	1.3

Liver-to-Body Weight Ratio

	**P value**	**Low VD**	**High VD**	**Low/High**
Wild type	0.721	8.721%	7.671%	1.137
Smad3$^{+/-}$	0.028	18.366%	6.248%	2.940

Lung Tumor Number

	**P value**	**Low VD**	**High VD**	**Difference**
Wild type	0.341	4.6	3.625	0.975
Smad3$^{+/-}$	0.324	4.5	6.188	-1.688
